# Evaluation of Clinical Outcomes of Laminectomy Versus Laminoplasty in Patients With Intradural Spinal Tumors: A Retrospective Observational Study

**DOI:** 10.7759/cureus.107687

**Published:** 2026-04-25

**Authors:** Bharatkumar R Dave, Mikeson Panthackel, Amritesh Singh, Ajay Krishnan, Shivanand C Mayi, Ravi Ranjan Rai, Mirant B Dave, Arjit Vashishtha, Yogenkumar Adodariya, Saurabh S Kulkarni, Mukesh Patel

**Affiliations:** 1 Spine Surgery, Stavya Spine Hospital and Research Institute, Ahmedabad, IND; 2 Orthopaedics, University College of Medical Sciences (UCMS) & Guru Teg Bahadur (GTB) Hospital, Delhi, IND; 3 Spine, Bhavnagar Institute of Medical Sciences (BIMS), Bhavnagar, IND; 4 Spine, Stavya Spine Hospital and Research Institute, Ahmedabad, IND; 5 Orthopedics, Mahatma Gandhi Medical College and Research Institute, Aurangabad, IND; 6 Neurological Surgery, Stavya Spine Hospital and Research Institute, Ahmedabad, IND

**Keywords:** intradural spinal tumors, laminectomy, laminoplasty, postoperative outcomes, spinal meningioma

## Abstract

Introduction

Intradural spinal tumors constitute a significant proportion of central nervous system neoplasms. Surgical resection remains the definitive treatment modality to achieve adequate tumor control and neurological preservation. While laminectomy has traditionally been the standard posterior approach for resection, it is associated with several postoperative complications. In recent decades, laminoplasty has emerged as an alternative surgical technique to address the shortcomings of traditional laminectomy. The optimal surgical approach that achieves complete tumor resection while minimizing postoperative morbidity and preserving spinal alignment continues to be debated. Our study aims to compare clinical outcomes between laminectomy and laminoplasty in patients with intradural spinal tumors.

Methods

This was a hospital-based, single-center retrospective observational study conducted on 100 consecutive patients with intradural spinal tumors equally divided into two groups: group A (laminectomy) and group B (laminoplasty). The aim of the study is to evaluate the clinical outcomes of laminectomy versus laminoplasty. Patient data were collected from the HMIS (Hospital Management Information System), and patient-related imaging was procured from PACS (Picture Archiving and Communication System). Data recorded included demographic data, operative parameters, functional outcomes, postoperative complications, and radiographic assessment. Statistical analysis was performed using IBM Corp. Released 2014. IBM SPSS Statistics for Windows, Version 20. Armonk, NY: IBM Corp. Quantitative data was represented as mean and standard deviation. The statistical significance was set at a p-value <0.05.

Results

A total of 100 patients were included (laminectomy = 50, laminoplasty = 50). Operative time was slightly longer with laminoplasty, while blood loss (p < 0.001) and hospital stay (p=0.003) were significantly lower. Neurological outcomes (Modified McCormick Scale) were equivalent in both groups at 12 months. Laminoplasty demonstrated significantly lower postoperative pain scores (VAS, p<0.001), fewer CSF leaks (2% vs. 12%, p=0.047), and fewer epidural hematomas. Overall complication rates were lower following laminoplasty.

Conclusion

Laminoplasty represents a secure and effective alternative to traditional laminectomy in terms of pain, hospital stay, intraoperative blood loss, and peri- and postoperative complications. The marginal increase in operative time is outweighed by the substantial reduction in perioperative morbidity and improved functional outcomes. Given these advantages, laminoplasty represents a safe, effective, and potentially preferable alternative to traditional laminectomy for adult patients with intradural spinal tumors, though these findings warrant further validation through prospective, multi-centric studies.

## Introduction

Intradural spinal tumors constitute a significant proportion of central nervous system neoplasms, with an estimated incidence ranging from 0.3 to 1.6 per 100,000 individuals annually. Among these lesions, the majority are classified as intradural extramedullary tumors, mainly consisting of schwannomas and meningiomas. Surgical resection remains the definitive treatment modality for optimal tumor control and neurological preservation [1,2].

Laminectomy has traditionally served as the standard posterior approach for intradural spinal tumor resection, providing wide exposure and satisfactory decompression of neural structures. However, this technique has been associated with several postoperative complications such as hematoma formation, iatrogenic spinal canal stenosis, progressive spinal deformity, prolonged postoperative pain, paraspinal muscle atrophy, cerebrospinal fluid leakage, and the need for revision surgery. The reported incidence of postoperative spinal deformity after laminectomy ranges from 10% in adults to 22-100% in pediatric populations, representing a significant concern for long-term functional outcomes [2-4]. Recent data indicate that complications such as cerebrospinal fluid leakage, infection, and hematoma formation occur more commonly in patients undergoing laminectomy than those undergoing laminoplasty [1].

Laminoplasty has recently emerged as an alternative surgical technique aimed at addressing the limitations of traditional laminectomy. It is a reconstructive approach involving either removal or retraction of the posterior elements followed by their anatomical restoration using various fixation methods, thereby theoretically preserving spinal stability and reducing postoperative complications. This approach allows tumor removal followed by restoration of the posterior spinal ring, providing a bony posterior roof for the spinal cord post-tumor excision. Despite mounting evidence in the literature supporting the potential advantages of laminoplasty, including maintenance of posterior spinal architecture, preservation of ligamentous structures, reduced epidural scarring, lower cerebrospinal fluid leakage rates, and reduced blood loss, the technique has not yet achieved universal acceptance within the neurosurgical community [1,2,5]. Some studies in the literature have demonstrated that laminoplasty significantly decreases the incidence of CSF leaks from incisional durotomies compared to laminectomy, likely due to the reconstruction of the posterior tension band and paraspinal muscle reattachment [5].

However, comparing the effectiveness of laminoplasty versus laminectomy in adult patients with intradural spinal tumors remains controversial, with inconsistent findings across published studies. The optimal surgical approach that achieves complete tumor resection while minimizing postoperative morbidity continues to be debated. Moreover, laminoplasty has been shown to offer shorter durations of postoperative pain and negate the financial and physical burden of additional instrumentation often required for fusion in unstable laminectomy cases [2-4].

We hypothesized that laminoplasty would yield superior perioperative safety, specifically a reduced incidence of postoperative complications and improved postoperative pain, compared to traditional laminectomy, without compromising neurological preservation or the extent of tumor resection.

Therefore, the primary objective of this study was to compare the overall incidence of postoperative complications between the two surgical techniques. The secondary objectives were to evaluate differences in perioperative parameters (operative duration, estimated blood loss, and length of hospital stay) and to assess long-term functional and neurological recovery.

## Materials and methods

A retrospective observational study was conducted on 100 consecutive patients with intradural spinal tumors who underwent surgical resection at a tertiary care spine institute. The study protocol was approved by the Institutional Ethics Committee (IEC), approval number SSHRI/CS/NS/Laminoplasty/BRD/83/12.25, and the requirement for informed consent was waived due to the retrospective nature of the analysis (CTRI/2025/12/099007).

Patients were equally divided into two groups based on the surgical technique employed and were followed up regularly for a period of 12 months. As a retrospective analysis, a convenience sample of consecutive eligible patients was utilized. Allocation of surgical technique was non-randomized and determined by the primary surgeon's discretion based on anatomical feasibility. Postoperatively, all patients were managed using standardized institutional perioperative protocols. To minimize observer bias, data extraction and functional outcome scoring were performed by an independent clinical researcher. Medical records were collected from the hospital database from January 2023 to December 2024. A) Laminectomy (Group A) (n = 50): Patients who underwent traditional laminectomy without reconstruction of the posterior elements. B) Laminoplasty (Group B) (n = 50): Patients who underwent en bloc laminoplasty followed by reconstruction of the posterior vertebral arch using suture fixation.

Eligibility criteria

Inclusion Criteria

The following category of patients was included in the study: a) patients aged 18 years and above. b) Histologically confirmed intradural spinal tumors, including schwannoma, meningioma, ependymoma, neurofibroma, hemangioblastoma, and astrocytoma. c) Tumor location: Intradural extramedullary, intradural intramedullary, or combined tumors situated at thoracic or lumbar spine levels. d) Primary surgical intervention consisting of either laminectomy or laminoplasty for tumor resection. e) Availability of complete preoperative and postoperative imaging (MRI, CT, or X-rays). f) Complete clinical records including demographics, operative data, and functional outcomes. g) Minimum postoperative follow-up duration of 12 months, h) Cases strictly amenable to gross total resection (GTR) with primary watertight dural suturing, without the need for complex dural reconstruction or dural patches.

Exclusion Criteria

The following category of patients were excluded from the study: a) patients with extradural or purely epidural tumors. b) Patients with metastatic spinal lesions. c) Dumbbell tumors requiring extensive facetectomy or anterolateral approaches. d) Patients who underwent multiple surgical interventions for recurrence or other spinal pathologies at different time points. e) Incomplete preoperative or postoperative clinical or radiographic data. f) Postoperative follow-up duration of less than 12 months. g) Patients undergoing concurrent fusion or instrumentation (e.g., pedicle screw fixation) at the time of tumor resection. h) Cases where primary dural closure could not be achieved and required the use of dural substitutes or patches.

Surgical technique

All surgeries were performed by a single experienced spine surgeon.

Laminectomy

A posterior midline incision was made on the back according to the level affected, followed by subperiosteal dissection of the paraspinal muscles. At the spinolaminar junction, bilateral cuts were made using an ultrasonic bone scalpel, and spinous processes along with the laminae were removed using a bone nibbler and Kerrison rongeurs to expose the dura. Facet joints were preserved. The dura was incised, and the resulting dural opening was extended via blunt dissection to carefully expose and isolate the tumor. After tumor resection and dural closure (assessed with the Valsalva maneuver), the wound was closed in layers without reconstruction of the posterior arch.

Laminoplasty

**Figure 1:**
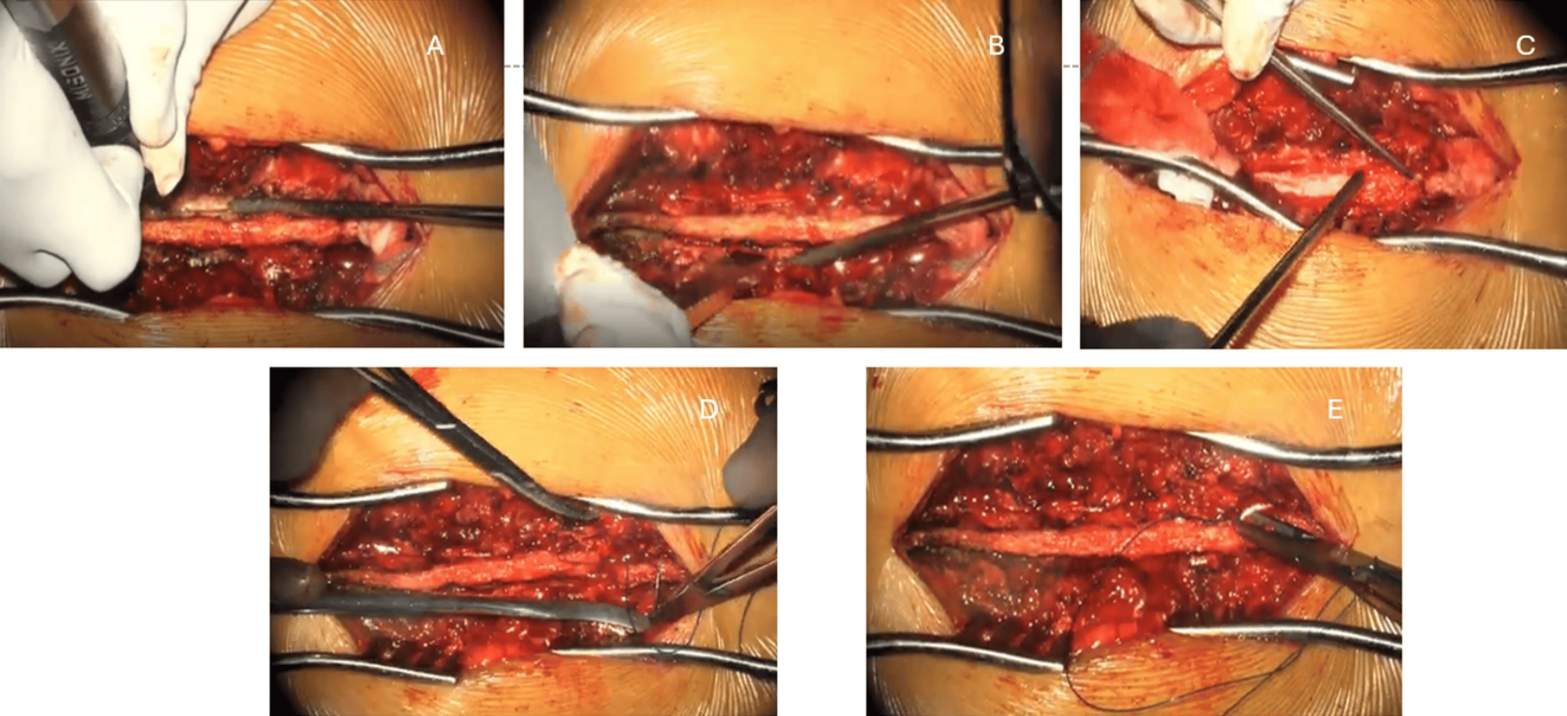
Intraoperative steps of the en-bloc laminoplasty technique. A: Achievement of bilateral laminar osteotomies using an ultrasonic bone scalpel (UBS). B: Confirmation of osteotomy completion using a specially designed osteotome; the instrument is gently rotated within the bone cuts to verify the mobility of the laminar segment. C: Retraction and stabilization of the laminar flap to one side using self-retaining retractors to allow for tumor exposure. D and E: Anatomical reconstruction of the posterior arch by securing the laminar flap with No. 1 Prolene sutures.

After exposure, bilateral cuts were made using an ultrasonic bone scalpel. The posterior arch (spinous process and laminae) was separated from either its cranial or caudal soft tissue attachment, elevated, and retracted to one side as a single flap. Following tumor removal and dural repair, the laminar flap was anatomically repositioned, and the separated attachment was secured using non-absorbable sutures (No. 1 Prolene) through the spinous process to restore the posterior spinal architecture. The above-mentioned steps are depicted in Figure 1.

**Figure 2:**
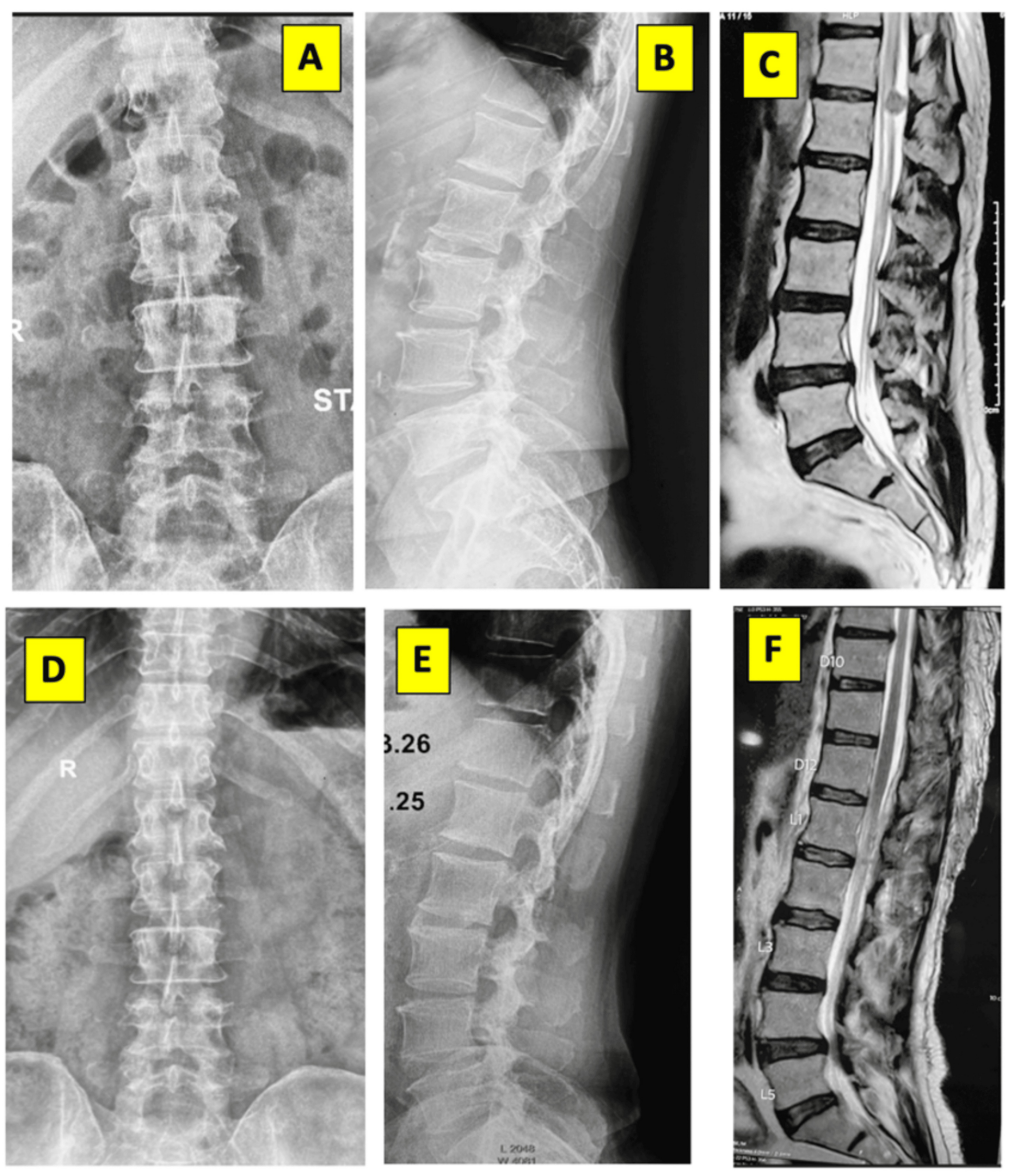
Preoperative and postoperative imaging of a dorsolumbar junction tumor. Illustration: (A, B) Preoperative X-rays, AP and lateral, demonstrating no significant structural abnormalities. (C) Preoperative sagittal MRI of the patient demonstrating an intradural extramedullary tumor at the D12-L1 level. (D, E) Immediate postoperative AP and Lateral X-rays. (F) Postoperative sagittal MRI confirming the complete gross total resection of the tumor.

A representative case from the laminoplasty cohort is illustrated in Figure 2.

Data collection and outcome measures

Patient data were procured from the HMIS (Hospital Management Information System) and radiological investigations from the PACS (Picture Archiving and Communication System), which included the following:

Demographic Data

Age, sex, body mass index (BMI), tumor histology, tumor location, and tumor type (intramedullary vs. extramedullary).

*Operative Parameters* 

The mean operative time (skin-to-skin), estimated blood loss, and length of hospital stay.

Functional Outcomes

Neurological status was assessed using the Modified McCormick Scale (MMS) [6]. Pain severity was evaluated using the Visual Analog Scale (VAS) [7]. Both scores were recorded in the preoperative period and at 3, 6, and 12 months of follow-up.

*Postoperative Complications (If Any)* 

Complications such as cerebrospinal fluid (CSF) leaks, surgical site infections, postoperative epidural hematomas, wound dehiscence, and spinal adhesions. Postoperative complications were strictly defined using established clinical criteria: surgical site infections (SSI) were classified according to the CDC/NHSN surveillance definitions [8], symptomatic postoperative epidural hematomas were defined by progressive neurological deficit correlating with compressive fluid collection on MRI [9], and clinical CSF leaks were identified by clear watery fluid in the drain, pseudomeningocele formation, or orthostatic headache [10].

Statistical analysis

Statistical analysis was performed using IBM Corp. Released 2014. IBM SPSS Statistics for Windows, Version 20. Armonk, NY: IBM Corp. variables were expressed as mean ± standard deviation, while categorical variables were expressed as frequencies and percentages. Such data was analyzed using the Chi-square test or Fisher’s exact test. Quantitative data were presented as mean and standard deviation and were analyzed using the independent Student’s t-test for normally distributed data or the Mann-Whitney U test for skewed data. Statistical significance was set at a p-value <0.05.

## Results

Intradural extramedullary tumors comprised 94% of cases (n=47) in the laminectomy group and 90% (n=45) in the laminoplasty group, whereas intramedullary ones constituted the rest. Schwannomas were the most common histopathological diagnosis, followed by neurofibromas and meningiomas in both groups, as depicted in Table 1. Tumor location was distributed across the thoracic spine (laminectomy 56%, n=28; laminoplasty 42%, n=21) and lumbar spine (laminectomy 44%, n=22; laminoplasty 58%, n=29).

**Table 1:** Distribution of the most common histopathological tumor types between the surgical cohorts: This table illustrates the frequency of the three most common histopathological diagnoses—schwannomas, neurofibromas, and meningiomas—encountered in the laminectomy (n = 50) and laminoplasty (n = 50) groups. Data are presented as absolute patient numbers (n) alongside their corresponding percentages (%). Schwannomas represented the most prevalent intradural tumor type across both surgical cohorts. Other histologies included ependymoma, hemangioblastoma, and astrocytoma.

Type of tumor	Laminectomy n(%)	Laminoplasty n(%)
Schwannoma	21 (42%)	21 (42%)
Neurofibromas	14 (28%)	18 (36%)
Meningiomas	12 (24%)	6 (12%)

The mean operative duration, estimated blood loss, and length of hospital stay are depicted in Table 2. Based on our strict eligibility criteria, Gross Total Resection (GTR) with primary watertight dural closure was successfully achieved in 100% of cases in both the laminectomy and laminoplasty cohorts. No patients in this study underwent subtotal resection (STR) or required the use of synthetic dural patches, ensuring that the intradural surgical phase was uniform across all 100 patients.

**Table 2:** Operative parameters recorded in both surgical groups: This table compares the perioperative parameters between the laminectomy and laminoplasty groups. Variables evaluated include mean operative duration (in minutes), mean estimated blood loss (in milliliters), and mean length of hospital stay (in days). Continuous data are presented as mean ± standard deviation (SD). Statistically significant differences were noted in blood loss, operative time (p < 0.001), and hospital stay (p = 0.003). The analysis was done using an independent student's t-test.

Parameter	Laminectomy (mean ± SD)	Laminoplasty (mean ± SD)	Test statistic	P - value
Mean operative duration	137.4 ± 10.01 minutes	149.4 ± 9.87 minutes	t=6.04	P <0.001
Mean blood loss	126.5 ± 14.1 mL	95.6 ± 12.6 mL	t=11.56	P <0.001
Mean hospital stay	6.3 ± 1.9 days	5.3 ± 1.4 days	t=3.00	p = 0.003

All patients completed the 12-month follow-up. The comparison of preoperative and postoperative functional outcomes and neurological status is presented in Table 3.

**Table 3:** Preoperative and postoperative pain and neurological scores. This table outlines the functional and neurological outcomes for both patient cohorts preoperatively and at the 12-month postoperative follow-up. Neurological status was evaluated using the Modified McCormick Scale (MMS), while pain severity was measured using the Visual Analog Scale (VAS). Data are reported as median values for the MMS and as mean ± standard deviation (SD) with corresponding ranges for the VAS scores. At the 12-month follow-up, patients undergoing laminoplasty demonstrated significantly lower postoperative pain scores compared with those undergoing laminectomy (VAS 2.2 ± 0.9 vs. 4.3 ± 1.1, t = 10.45, p < 0.001, independent Student’s t-test).

Score	Laminectomy	Laminoplasty	Test statistic	P - value
MMS preoperative (neurological status) (median values)	2	2	N/A	N/A
MMS postoperative at 12 months (neurological status) (median values)	2	2	N/A	N/A
VAS score preoperative {mean ± SD (range)}	6.1 ± 1.1 (4 – 9.1)	6.1 ± 1.2 (3.5 – 8.7)	t=0.00	0.94
VAS score postoperative at 12 months {mean ± SD (range)}	4.3 ± 1.1 (1.7 – 7)	2.2 ± 0.9 (1 – 5)	t=10.45	< 0.001

Cerebrospinal fluid (CSF) leakage was the most common postoperative complication, with detailed complication data summarized in Table 4. Among the laminectomy patients who experienced a CSF leak, two required carbonic anhydrase inhibitor therapy, while the remainder resolved spontaneously. The single case of CSF leakage in the laminoplasty group resolved spontaneously with conservative management. All infections were managed conservatively with appropriate antibiotic therapy without requiring reoperation. None of the cases with hematoma required emergency decompression and were managed conservatively with serial neurological assessments. No cases of wound dehiscence occurred in either group. Deep vein thrombosis and pulmonary embolism were not encountered in either cohort. A strong trend toward a higher total postoperative complication rate was observed in the laminectomy group (n=12, 24%) compared to the laminoplasty group (n=2, 4%), though this did not reach statistical significance (p = 0.062, Fisher's exact test).

**Table 4:** Characteristics of postoperative complications This table details the incidence and characteristics of postoperative complications encountered in the laminectomy and laminoplasty groups. Categorical data, including the number of cases with cerebrospinal fluid (CSF) leakage, superficial surgical site infections, and postoperative epidural hematomas, are presented as absolute frequencies (n). Continuous variables detailing the average daily volume and duration of CSF leaks are expressed as mean ± standard deviation (SD). Statistical analysis was done using Fisher's exact test. A statistically significant difference was observed in the incidence of CSF leakage between the two cohorts (p = 0.047).

Parameter	Laminectomy	Laminoplasty	Test statistic	P -value
Cases with CSF leakage (n)	6	1	N/A	0.047
Average amount of CSF leak (mean ± SD)	141 ± 35 mL/day	49 mL/day	-	-
Duration of CSF leak (mean ± SD)	5.8 ± 1.3 days	4 days	-	-
Cases with Superficial surgical site infection (n)	2	1	N/A	0.99
Cases with Postoperative epidural hematoma (n)	4	0	N/A	0.121

To further evaluate the impact of surgical technique on postoperative complications while accounting for baseline demographic differences, a multivariate logistic regression model was performed. When adjusting for BMI, surgical technique (laminectomy vs. laminoplasty) trended toward a higher risk of complications but did not reach statistical significance as an independent predictor (OR = 3.53; 95% CI: 0.84-14.83; p = 0.085). Similarly, BMI did not independently predict complications in this adjusted model (p = 0.156). 

## Discussion

The surgical management of intradural spinal tumors is a delicate balance between achieving a gross total resection and maintaining long-term structural integrity of the vertebral column [11]. Laminectomy is regarded as the standard workhorse approach, which provides adequate visualization and careful as well as safe manipulation of the spinal cord [12]. However, the subsequent loss of the posterior tension band that often comes as a consequence leads to a cascade of biomechanical failures [12].

Our retrospective analysis was a hospital-based, single-center observational study. Our sample comprised 100 patients who satisfied the selection criteria and were equally divided into two groups: Group A (laminectomy) and Group B (laminoplasty). The aim of the study was to evaluate the clinical and functional outcomes of laminectomy versus laminoplasty in patients with intradural spinal tumors.

In our study, the mean operative time was longer in the laminoplasty group compared to the laminectomy group (149.4 ± 9.87 vs. 137.4 ± 10.01 minutes). This increase can be attributed to the time required for secure fixation of the repositioned laminae and meticulous suturing to restore posterior ligamentous continuity. We observed a statistically significant reduction in the mean estimated blood loss within the laminoplasty group (p < 0.001). This observation is likely due to the more contained nature of the dissection, the preservation of posterior bone and ligamentous structures, and the avoidance of extensive dissection inherent to the laminoplasty technique. Reduced blood loss corresponds to less postoperative swelling and a clearer surgical field, which is essential when working around the spinal cord [12]. This reduction in intraoperative blood loss observed in our laminoplasty group also suggests that preserving the laminar arch reduces the exposed cancellous bone surface area, a finding which is corroborated by Sun et al., who also documented a statistically significant reduction in blood loss (p < 0.00001) in laminoplasty cohorts, further validating the physiological advantages of the technique [12].

Patients in the laminoplasty group experienced a statistically significantly shorter hospital stay compared to those in the laminectomy group (5.3 ± 1.4 days versus 6.3 ± 1.9 days; p = 0.003). This clinically meaningful reduction was attributed to lower postoperative pain, a decreased incidence of CSF leakage, and the early achievement of mobilization milestones [13].

Another objective of our study was to evaluate postoperative pain, which contributes significantly to morbidity and remains a primary concern for patients. The techniques of laminoplasty particularly prioritize the anatomical reattachment of the posterior musculature [14]. Patients undergoing laminoplasty reported significantly lower pain scores at 12 months compared to laminectomy patients (VAS 2.2 ± 0.9 vs. VAS 4.3 ± 1.1; p < 0.001). This improvement in long-term pain scores is likely due to the prevention of the post-laminectomy membrane. Singh et al., in their study, suggested that the repositioned lamina acts as a mechanical barrier, thereby preventing the adherence of paraspinal muscles to the dura and minimizing the chances of epidural fibrosis, which is a known generator of neuropathic pain [1]. Although laminoplasty provides superior stability, post-operative neck and shoulder pain remains a significant complication in 60% of cases, primarily due to the detachment of the deep extensor muscles [15].

Once a tumor is removed, the spinal cord needs a controlled environment to re-expand and re-perfuse [16]. After laminectomy, the spinal cord is left exposed and vulnerable to the formation of a post-laminectomy membrane [1], a dense scar tissue that can constrict the dural sac. By reconstructing the bony arch, laminoplasty gives the spinal cord the space it needs to heal while maintaining a bony shield that prevents the cord from tethering against posterior soft tissues. This was corroborated in a recent meta-analysis of over 1,000 cases, finding an effective recovery rate of 88.28% for laminoplasty compared to 71.58% for laminectomy [12]. Lee et al. emphasized that while preoperative status is the strongest predictor of outcome, the surgical technique protects that recovery from secondary deterioration [17]. In our analysis, we observed comparable neurological outcomes between the two groups, with a median Modified McCormick Score of 2 in both cohorts, though the differences were not statistically significant.

Our study demonstrated a better safety profile for laminoplasty, with a strong trend toward a lower total complication rate (4%) compared to the laminectomy group (24%). Most notably, the incidence of cerebrospinal fluid (CSF) leakage was significantly lower in the laminoplasty group (n=1, 2% vs. n=6, 12%; p = 0.047). The retrospective review on incidental durotomy emphasizes that managing a dural tear is fundamentally easier with a bony roof present acting as a mechanical backstop [18]. The restoration of the posterior bony arch eliminates dead space and provides structural support to the dural closure, preventing the formation of pseudomeningoceles. This is supported by Singh et al., who reported a leak rate of only 3.8% in laminoplasty patients versus 26.7% in laminectomy patients [1]. By keeping the posterior space closed, we significantly reduced the risk of seroma or hematoma formation, which is often a precursor to infection. Consequently, the lower incidence of complications within our laminoplasty group likely contributed to a significantly shorter hospital stay, a clinical finding corroborated by Byvaltsev et al., who cited this as a key advantage of the laminoplasty technique [5]. Although laminoplasty is traditionally viewed as a prophylactic measure against long-term biomechanical instability, our findings demonstrate that the anatomical reconstruction confers substantial acute perioperative benefits, specifically by reducing the incidence of cerebrospinal fluid leaks and postoperative epidural fluid accumulation.

While our initial analysis demonstrated a strong trend toward a lower total complication rate for laminoplasty, our multivariate model adjusting for BMI did not reach statistical significance. We attribute this mathematical limitation directly to the overall excellent safety profile observed in our cohort. With only 11 total complication events occurring across 100 patients, regression models inherently lack the statistical power (events-per-variable) required to definitively isolate the independent effect of surgical technique from confounding patient factors. Furthermore, sub-analysis of complication rates among specific tumor histologies was precluded by these low event frequencies. Nonetheless, the clinical reduction in morbidity remains highly relevant and supports laminoplasty as a tissue-sparing alternative.

The preservation of the posterior ligamentous complex is the biomechanical cornerstone of laminoplasty. A cadaveric study in 2009 concluded that a laminectomy significantly increases sagittal flexibility, which in turn destabilizes the segment and shifts the center of rotation [19]. Though our study focused on clinical outcomes, we must acknowledge that post-operative deformity is the long game of posterior surgery. Laminectomy patients face a three-fold higher risk of developing progressive spinal deformity compared to laminoplasty [3]. It is important to state that our study did not explicitly track long-term radiographic sagittal parameters as a primary outcome. However, based on the biomechanical evidence provided in the literature, the avoidance of kyphosis is a primary benefit of the motion-preserving approach [4,11]. Further prospective studies with 5-10 year radiographic follow-up are necessary to fully quantify these benefits.

Despite these promising results, there are several limitations to this study that must be addressed and acknowledged. First, the retrospective, observational design introduces the potential for selection bias, as the choice of surgical technique was not randomized. Variations in tumor characteristics or surgeon preference could influence outcomes. Second, all procedures were performed by a single surgeon at a single institution; while this ensures technical consistency and eliminates inter-operator variability, it may limit the generalizability of the results to other centers with different surgical protocols or experience levels. Third, the non-randomized, retrospective nature of this study resulted in baseline demographic and clinical imbalances between the two cohorts. Specifically, the laminectomy group demonstrated a significantly higher mean BMI (p = 0.026), alongside variations in tumor histopathology, including a higher proportion of meningiomas. Because elevated BMI and specific tumor characteristics (such as vascularity and dural adherence) are established independent risk factors for perioperative complications, these imbalances act as confounding variables. Although we attempted to control for this mathematically using multivariate logistic regression, the overall low complication rate in our study (14 total events) limited the statistical power necessary to completely remove the effect of surgical technique from these baseline differences. Future prospective, multi-centric studies stratified for BMI and tumor characteristics are necessary to overcome these specific epidemiological limitations. Furthermore, 12 months is insufficient to definitively capture late-onset structural instability or long-term progressive spinal deformity. Future prospective, multi-centric studies with long-term radiographic follow-up (5-10 years) are necessary to fully quantify the biomechanical durability of this technique.

## Conclusions

In conclusion, this retrospective cohort study suggests that en-bloc laminoplasty is a safe, effective, and tissue-sparing alternative to traditional laminectomy for the resection of uncomplicated intradural spinal tumors. Both techniques allow for excellent oncological clearance and neurological preservation. However, the anatomical reconstruction provided by laminoplasty is associated with a clinically meaningful reduction in acute perioperative morbidity, particularly regarding cerebrospinal fluid leakage. While these acute benefits appear to justify the marginal increase in operative time, future prospective, randomized trials with long-term radiographic follow-up are required to definitively establish the technique's overarching biomechanical superiority.
